# Are ^61^Cu, ^64^Cu, and ^67^Cu Really Happening?

**DOI:** 10.2967/jnumed.126.272416

**Published:** 2026-07

**Authors:** Richard Zimmermann

**Affiliations:** Chrysalium Consulting, Lalaye, France; MEDraysintell/NucAdvisor, Paris, France; and Oncidium Foundation, Mont-Saint-Guibert, Belgium

To date, nuclear medicine experts have investigated 5 copper isotopes: ^60^Cu, ^61^Cu, ^62^Cu, ^64^Cu, and ^67^Cu. Together, these exhibit radiophysical properties that cover both diagnostic and therapeutic applications, encompassing nearly all types of emission—positrons, γ-rays, conversion electrons, and x-rays, as well as β-particles and Auger electrons for therapy—with the exception of α-particles. These represent a compelling example of true radiotheranostics, even though currently available diagnostic or therapeutic drug pairs achieve efficiency without relying on isotopes of the same element (1).

The purpose of this document is to highlight the industrial and commercial advantages of copper isotopes and identify the production challenges that must be addressed to ensure long-term access and reliability for developers.

Because of their very short half-life and the lack of industrial interest in the development of production technologies, the imaging isotopes ^60^Cu (half-life, 23.7 min) and ^62^Cu (half-life, 9.7 min) will not be discussed in detail here.

## DO WE NEED ALTERNATIVE RADIONUCLIDES?

^99m^Tc in SPECT imaging, along with ^18^F and ^68^Ga in PET imaging, are used in over 95% of all diagnostic applications. Similarly, therapeutic applications predominantly use molecules labeled with ^131^I and ^177^Lu. A new frontier is emerging with α-emitters, including ^225^Ac (2), ^212^Pb (3), and ^211^At (4). The introduction of new radionuclides is justified when they demonstrate clear advantages, such as superior physical or chemical properties, easier accessibility (lower production costs, simplified synthesis, or readily available starting materials), marketing benefits (improved dosimetry, reduced radioactive waste) or the ability to circumvent existing patents. The key question is: in which areas could copper isotopes provide advantages over the established options?

True theranostic pairs (i.e., isotopes of the same element used for both diagnosis and therapy) play a crucial role in the development of radiotherapeutics. However, in practical applications, potential responders to therapy can also be identified using different radionuclides. Currently, theranostic pairs, such as ^18^F/^177^Lu or ^68^Ga/^177^Lu, are routinely used for this purpose. This implies that the introduction of a new imaging agent is justified only if no equivalent is already available on the market or if it offers a genuine economic advantage (greater accessibility, lower cost) compared with approved agents, not merely for marginal improvements in imaging quality. In the future, the selection of potential responders may even be achieved through a simple blood test.

In the radiopharmaceutical field, resources and funding should be prioritized for the exploration of new biologic mechanisms and novel targets, which will require the development of new tracers for clinical use. Research and development efforts in established areas should be regarded primarily as academic pursuits, as they are unlikely to result in new marketed imaging agents.

Against this backdrop, we can now assess the advantages offered by the 3 copper isotopes of interest. To date, more than 50 drug candidates associated with ^64^Cu and 7 with ^67^Cu are in development (5). The properties of these 3 isotopes are summarized in Table 1.

**TABLE 1.** Properties of ^61^Cu, ^64^Cu, and ^67^Cu*

Property	^61^Cu	^64^Cu	^67^Cu
Use	PET imaging	PET imaging, therapy	Therapy
Half-life (h)	3.34	12.70	61.83
Main decay mode	β^+^/EC	β^+^/EC and Ae	β^−^
γ (keV)	283 (13); 656 (10)		185 (49); 93 (16)
β^+^/EC (keV)	524 (51); 2238 max/EC (16)	278 (17); 1675 max/EC (46)	
β^−^ (keV)		191 (38); 580 max	121–189 (99); 562 max
Ae yield per disintegration with energy threshold		0.79 > 6 keV	1.2 > 7 keV
Preferred production route	Cyclotron, 8–12 MeV	Cyclotron, 11–20 MeV H; accelerator, 40 MeV D	Accelerator, 30 MeV H; photonuclear, 40–70 MeV

* All decay in a single step in stable isotopes.

EC = electron capture; Ae = Auger electron; D = deuteron; H = hydrogen.

Numbers in parentheses represent percent yield.

## ^64^Cu

^64^Cu is the most widely used radioisotope in this family (6,7). Its half-life of 12.7 h offers a significant advantage over other PET imaging agents by expanding the potential distribution area, despite the resulting reduced image quality caused by poor photon statistics. The longer half-life also supports the use of vectors requiring extended biologic residence time, such as antibodies. Curium is currently the first and only company to hold marketing authorization for a ^64^Cu-labeled tracer, specifically ^64^Cu-DOTATATE. This molecule is available in the United States and may soon be introduced in Europe and Asia. Its development was driven not only by the need to bypass patents covering ^18^F- and ^68^Ga-labeled analogs but also by the goal to ensure accessibility to remote sites. In parallel, Curium is pursuing clinical investigation of ^64^Cu-PSMA analogs, while more than 30 other ^64^Cu-labeled molecules are in clinical development.

^64^Cu is currently produced by irradiating a ^64^Ni target with a 10–13 MeV proton beam via the [^64^Ni(p,n)^64^Cu] reaction. After approximately 10 h of irradiation, yields can reach approximately 160 GBq per batch, corresponding to roughly 740 GBq per week. A second batch per day is impractical because of hospital operating hours; however, shorter runs—2 daily irradiations of about 6 h each, yielding 74 GBq per run—would achieve nearly the same weekly production capacity. Using a 200-MBq dose at calibration, and accounting for 1 half-life equivalent lost during labeling, handling, and transport, this output translates to approximately 2500 patient doses per week and more than 125,000 doses annually.

Alternative production routes, such as [^64^Ni(d,2n)^64^Cu] or [^64^Zn(d,2p)^64^Cu], are more efficient but require cyclotrons with deuteron beams. The highest reported capacity comes from 40-MeV accelerators using the ^66^Zn(d,α)^64^Cu reaction, a technology developed by Nusano. The company claims a potential annual production capacity of 925 TBq at end of beam (814 MBq/µA/h), equivalent to more than 2 million doses.

Curium requires a high number of doses and relies on high-current cyclotrons centrally installed at its U.S. facilities, with sufficient capacity to meet future demand. Expansion of this network would be necessary only to enter new markets (e.g., Europe, Asia) or support the launch of additional tracers.

Although the target material (^64^Ni) is relatively expensive ($50,000–$60,000 per gram), its recovery is straightforward, making the overall economics highly favorable. Companies in this field report having secured stocks or reliable access to ^64^Ni. The production of more than 100,000 doses annually from a single site is unnecessary, as this would exceed demand within feasible transportation limits. In practice, 2 or 3 cyclotrons are sufficient to cover the United States or Europe, rendering investment in production tools for a ^64^Cu-labeled drug highly affordable. In short, access to ^64^Cu in the near future should no longer be considered uncertain.

## ^61^Cu

More recently, ^61^Cu has attracted interest as an alternative to ^64^Cu, primarily because of its shorter half-life. Several new cyclotron production routes have been investigated to generate large amounts of ^61^Cu: [^61^Ni(p,n)^61^Cu], [^60^Ni(d,n)^61^Cu], or [^nat^Ni(d,xn)^61^Cu] or [^64^Zn(p,α)^61^Cu]. The [^62^Ni(p,2n)^61^Cu] nuclear reaction (8), developed at the University Hospital Zurich by Swiss Nuclides GmbH (now Nuclidium AG) appears to be the most efficient pathway for larger-scale industrial applications.

However, a dedicated network of ^61^Cu-producing cyclotrons does not yet exist, and reliance on the current large-scale cyclotron infrastructure is unrealistic for the reasons outlined below. Consequently, ^61^Cu developers must establish their own network if they aim to reach a market comparable in size to that of ^18^F-labeled drugs. Nuclidium, in partnership with GE HealthCare, has announced plans to create such a network. The first six ^61^Cu-dedicated European sites, as well as 6 in the United States, are expected to be built within the next 3 y, opening an entirely new area of research in this field. With a production capacity of 100–115 GBq every 200 min and 4 batches per day, assuming an average patient dose of 120 MBq (equivalent to approximately 370 MBq at end of beam), a single cyclotron could produce up to 1000 doses per day. In theory, this network would be sufficient. However, in practice, a doubling in size will be necessary to ensure coverage of remote sites. Overall, the establishment of roughly 24 or more centers would represent an investment of $150,000,000–$200,000,000, a realistic figure and significantly lower than the investment for the ^18^F infrastructure.

## ^67^Cu

At the level of therapeutics, any improvements in efficacy (e.g., new indications, better clinical outcomes, lower doses, fewer administrations), as well as advances in cost efficiency, final drug price, accessibility, sustainability, clinical waste management, will be welcomed by the nuclear medicine community, patients, and regulatory authorities. On this basis and among the relevant β-emitters, even though ^67^Cu shares similarities with ^177^Lu and ^161^Tb, this radionuclide and its associated radiopharmaceuticals may offer a distinct advantage, even at equal therapeutic efficacy, because of its shorter half-life (2.6 d vs. 6.7 d for ^177^Lu and 6.9 d for ^161^Tb). In addition to its β-emission, ^67^Cu also produces Auger electrons, which may confer a therapeutic benefit. This dual β-electron/Auger electron emission profile, similar to that of ^161^Tb, could confer an advantage over ^177^Lu for this specific reason; however, this potential advantage remains to be demonstrated.

Clarity Pharmaceuticals has long been the leader in the development of ^67^Cu-labeled radiotherapeutics. Its first candidate, ^67^Cu-SAR-bisPSMA, has advanced to a phase 3 clinical trial in metastatic castration-resistant prostate cancer, with completion expected by the end of 2026. To secure access to ^67^Cu, Clarity partnered with several manufacturers, including NorthStar, which now produces ^67^Cu using an electron accelerator (Rhodotron-HE-IBA) via the [^68^Zn(γ,p)^67^Cu] reaction and granted exclusivity to Clarity. Nusano, meanwhile, claims the ability to produce up to 120 TBq of ^67^Cu annually using its proprietary technology, which achieves yields of 48 MBq/µA/h, with targets irradiated using a 3-mA current.

Although some ^64^Cu contaminates the batch, its activity decays below the threshold during transport. The first doses are expected to be distributed by the first quarter of 2026. With an average patient dose of 7.4 GBq at end of beam, this capacity translates to approximately 15,000 doses per year, doses for approximately 5000 patients. However, this figure remains far below the 100,000-patient target for a large-scale population.

All companies currently developing ^61^Cu and ^64^Cu tracers report that their program already includes, or will be expanded to incorporate, ^67^Cu-labeled therapeutics. As a result, demand for ^67^Cu is expected to increase substantially in the near future.

## IS THERE A NEED FOR NEW COPPER-LABELED PET TRACERS?

With the exception of ^68^Ga, which can be produced via generators, commercialized PET tracers, primarily labeled with ^18^F or ^68^Ga, require a network of cyclotrons, the density of which is inversely related to the radionuclide’s half-life. Rough estimates incorporating transportation distances, half-life, and patient accessibility suggest that approximately 110 fully equipped cyclotrons are needed to supply ^18^F-labeled tracers across the 3 major markets (North America, Europe, and Japan).

Although the global cyclotron base has now reached 1650 units, the number of sites relevant to industry is lower when experimental and research tools, high- or low-energy machines, outdated equipment, and non–Good Manufacturing Practice centers are excluded. In practice, fewer than 950 cyclotrons can be considered true PET radiopharmaceutical manufacturing facilities. The cyclotrons were originally built to produce ^18^F-FDG. With the recent introduction of new ^18^F-labeled tracers (e.g., for neurology and prostate cancer), most sites are already operating at full capacity with marketed molecules. Furthermore, the ongoing transition of ^68^Ga production from generators to cyclotrons will place additional demands on the remaining available capacity. Given the limitations in space, staff, and laboratory infrastructure at each site, a fully equipped facility can realistically produce, at large scale (i.e., the same batch frequency as ^18^F-FDG per day), no more than 2 or 3 additional molecules alongside ^18^F-FDG.

Another important factor must be considered: the new generation of diagnostic agents, including all ^18^F- and ^68^Ga-labeled drugs, are protected by patents, and only licensed centers will be permitted to produce these molecules. The pharmaceutical companies that own these agents will retain control over the production network and prohibit their licensees from contracting with organizations targeting the same patient population. In other words, given the number of ^18^F- or ^68^Ga-labeled molecules expected to reach the market over the next decade, the existing network will soon be saturated. Companies developing ^18^F-labeled tracers that are not commercialized by 2030 will need to consider investing in their own production infrastructure. For a fluorinated tracer, such an investment could exceed $600,000,000 per molecule.

By contrast, ^61^Cu offers a potential advantage. With a half-life slightly longer than that of ^18^F (3.3 h versus 1.8 h), ^61^Cu would require a smaller production network. Rough estimates suggest that approximately 60 centers could be sufficient to reach the same patient population, representing a significant logistic benefit for this radionuclide. With a half-life of 12.7 h, the infrastructure required for ^64^Cu-labeled drugs (about 12 centers) aligns production investment with the standard costs of conventional drug development.

The absence of a strong γ-emitter within the copper isotope family means that these isotopes cannot serve as an alternative to the unique, inexpensive, and highly effective SPECT imaging agent ^99m^Tc. Unfortunately, PET agents are not yet viable solutions in regions where PET imaging infrastructures (cyclotrons and cameras) are missing, a situation that will probably not improve for the next 2 decades. Patient selection for therapeutics that can easily be imported in these countries will primarily rely on ^99m^Tc-labeled agents. This limitation is underscored by the distribution of imaging equipment. Globally, the ratio of PET to SPECT cameras is approximately 1:4. In South America, Africa, and most Asian countries, including India, the disparity is even greater, with a ratio closer to 1:20 (5).

## THERAPY: TAKING ADVANTAGE OF AUGER ELECTRON EMISSION?

The strong Auger electron emission of ^64^Cu is an important factor that has encouraged developers to leverage this property for therapeutic applications. However, high efficacy with Auger electrons can be achieved only if the molecule crosses the cell membrane, allowing it to reach the cell nucleus and DNA as closely as possible.

Free copper in the form of chloride salt can be used to image cell proliferation, as human copper transporter 1 is generally overexpressed in several forms of cancer (9). ^64^Cu-chloride is explored in phase 3 clinical trials for PET imaging of patients with PSMA-negative biochemical recurrence and at high risk of metastases. For years, ACOM has promoted the development of ^64^Cu-chloride as an alternative to ^18^F-FDG and ^18^F-fluorocholine. Unlike receptor-targeting agents, copper participates in cell proliferation and signaling processes, including DNA replication. Three companies (ACOM, LinqMed, and Oncurie) are actively pursuing this approach with ^64^Cu-labeled molecules, advancing in phase 2 and 3 clinical trials. In Europe, a phase 2 trial with ^64^Cu-chloride is being planned for patients with recurrent glioblastoma, an indication for which ACOM has obtained orphan drug designation. If this trial proves successful, ^64^Cu could be effective across a wide range of fast-growing tumor types, potentially broadening its clinical applications significantly. The same progresses are made by LinqMed with ^64^Cu-ATSM (10) in brain tumors.

Regardless of the success of this development, access to the radionuclide would require a 20-fold increase in production capacity to meet market demand, based on an average injected human dose of 4.8–7.4 GBq. In such a scenario, the high-capacity tools described above would prove valuable, and the investment would be justified.

## CONCLUSION AND FUTURE OUTLOOK

Within the radionuclide armamentarium, the copper family offers unique characteristics. Three copper isotopes show strong potential, each requiring specific adaptations. ^64^Cu, currently used to circumvent existing patents, appears to be an ideal radionuclide for enabling access to PET imaging agents at relatively low investment levels, eliminating many of the constraints and costs associated with the development of ^18^F- and ^68^Ga-labeled analogs. A substantial number of ^64^Cu-based imaging agents are now under development. ^64^Cu, in the forms of copper chloride and ^64^Cu-ATSM, is being further investigated and developed as a therapeutic agent, capitalizing on its Auger electron emission.

^61^Cu is emerging as an alternative to ^68^Ga, offering a longer half-life. Its development is expected to progress now that the first production network investment has been secured. If the market price of ^61^Cu-based imaging agents (for already-explored indications) proves competitive with existing likely generic tracers at the time to market, these agents could capture a meaningful share of the market. Nevertheless, ^61^Cu will continue to face strong competition from ^64^Cu, and indirectly with ^18^F and ^68^Ga.

^67^Cu is expected to reach the market, provided that the molecules currently in phase 3 trials, will demonstrate superiority over the marketed ^177^Lu-labeled analogs. With comparable therapeutic efficacy, ^67^Cu could serve as an alternative to ^177^Lu, offering a significant advantage in terms of half-life and improved management of human radioactive waste. Such success would likely stimulate interest in the development of additional molecules labeled with ^67^Cu. However, at that point, financial participation from third parties will be necessary to share or secure the investment required for expanding the production network. ^67^Cu will continue to face strong competition from ^177^Lu and, in the near future, from ^161^Tb.

## DISCLOSURE

No potential conflict of interest relevant to this article was reported.
